# Optimization of ultrasonic Bath and cold plasma pre‐treatments in the spearmint essential oil isolation process

**DOI:** 10.1002/fsn3.3224

**Published:** 2023-01-11

**Authors:** Jalil Moradi‐Sadr, Mohammad‐Taghi Ebadi, Mahdi Ayyari, Hamidreza Ghomi

**Affiliations:** ^1^ Department of Horticultural Sciences Tarbiat Modares University Tehran Iran; ^2^ Laser and Plasma Research Institute Shahid Beheshti University Tehran Iran

**Keywords:** cold plasma, hydro‐distillation, optimization, spearmint, ultrasonic

## Abstract

Spearmint essential oil (SEO), one of the economically valuable natural products, has special importance in the food, pharmaceutical, and perfumery industries due to its antifungal, antioxidant, and enzyme inhibitory properties. In this study, we optimized and evaluated the effect of three pre‐treatments on the extraction of SEO for quantity and quality: ultrasonic bath (UB), water to material ratio‐ultrasonic bath (W/M‐UB), and cold plasma‐ultrasonic bath (CP‐UB). Three experiments were designed using the central composite design (CCD) of response surface methodology (RSM). Experimental treatments included UB temperature (30–80°C) and time duration (1–30 min), cold plasma (CP) power (15–24 kV), and water/material ratio (10–40). Then, SEOs were extracted by hydro‐distillation using the Clevenger apparatus. The results showed that SEO yield in the optimal conditions of treatments was 119.7%, 206.6%, and 155.7% higher in UB, W/M‐UB, and CP‐UB pretreatments respectively, in comparison to control sample and optimized conditions were UB temperature: 37.3°C and UB time: 5.2 min at UB treatment, 33.9 of W/M ratio, 69.9°C of UB temperature and 6.9 min of UB time at W/M‐UB treatment and CP power: 22.176, UB temperature: 40.135 and UB time: 24.122 at CP‐UB treatment. Oxygenated monoterpenes were also higher in the essential oils (EOs) of all three treated plant materials. In conclusion, the SEO extraction yield improved by the application of the pretreatments in optimized conditions.

## Graphical abstract

The aim of this study was to increase the extraction efficiency of essential oil. Treatments were ultrasonic bath, water to material ratio, and cold plasma. Increasing the time of ultrasonic bath and cold plasma power led to a higher yield. Treatments with ultrasonic bath temperature and water/material ratio inversely affected the yield. The treatments raised the percentage of oxygenated monoterpenes slightly.

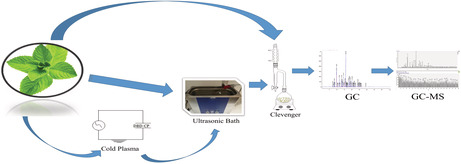

## 1 INTRODUCTION

Medicinal plants are one of the main functional foods. A foodstuff is considered to be functional in addition to its nutritional properties when it has a positive effect on health, for example in illness prevention or cure. There are about 236 genera and more than 6000 species in the Lamiaceae family. Many species of the family are cultivated worldwide and most of them are aromatic plants. The mint family (Lamiaceae) is also one of the main functional food sources (Birch & Bonwick, 2019; Carović‐StanKo et al., 2016).

Genera *Mentha* consists of 42 species, 15 hybrids, and 100 of subspecies, varieties, and cultivars (Lawrence, 2007) and is one of the most widely employed genera among Uzbek traditional remedies (Mamadalieva et al., 2017). Within the genus, 11 hybrids have been produced by crossing five species: *M. aquatica* L., *M. longifolia* L., *M. arvensis* L., *M. suaveolens*, and *M. spicata* L. (Spencer et al., 1993).

Spearmint (*Mentha spicata* L.) is an annual plant from the Lamiaceae family and is widely distributed in Africa, temperate Asia, and Europe (Kumar et al., 2011). Spearmint essential oil (SEO) is effective in the treatment of Alzheimer's disease, drug‐resistant infections, dermatophytosis, and obesity (Ali‐Shtayeh et al., 2019). SEO has anti‐aflatoxigenic, antifungal, and insecticidal properties. Because of its useful properties, it has a great value in the food industry (Kedia et al., 2014). Carvone, limonene, linalool, 1,8‐cineole, and dihydrocarveol are the main constituents of SEO (Mokhtarikhah et al., 2020).

Worldwide, standardized SEO is one of the most useful additives in the food, pharmaceutical, cosmetic, and hygiene industries. In 2019, SEO (HS code: 330125) comprised of 20% of the total market value for essential oils (EOs; 1.3 billion dollars), whereas the market values for orange and peppermint EOs were 8% and 3%, respectively (Essential Oils (HS: 3301)) *Product Trade, Exporters and Importers | OEC ‐ The Observatory of Economic Complexity*, (2020).

In recent years, various treatments were studied for EO extraction regarding their effect on chemical composition and the yield of EOs. One of the treatments evaluated by many researchers was the ultrasonic pretreatment (da Silva Moura et al., 2020; Liu et al., 2019; Priyadarshi et al., 2021; Zorga et al., 2020). Another attractive treatment experimented by a large number of researchers is the microwave pretreatment or microwave assisted extraction (Drinić et al., 2020; Radzi & Kasim, 2020; Tran et al., 2018) and etc. Other methods such as Ohmic‐assisted hydro‐distillation (HD; Gavahian et al., 2018), enzyme treatment (Morsy & Hammad, 2021), cold plasma (CP; Shokoohi et al., 2022), and salted distillation water treatment (Dao et al., 2020) were also evaluated in recent years for EO extraction from many medicinal and aromatic plants (Table 1).

**TABLE 1:** Microwave, ultrasonic and Ohmic‐assisted hydro‐distillation and also cold plasma, enzyme and NaCl treatments in recently reported studies

Plant name	Extraction method	Pretreatment	Optimized parameters	Yield improvement (%)	Reference
*Mentha spicata* L.	HD^a^	Cold plasma	Exposure time, voltage value	1	Rezaei et al. (2021)
*Mentha spicata* L.	MAHD^b^	_	Microwave power, process time	0.3	Shankar and Natarajan (2022)
*Iberis amara* (seeds)	UAHD^c^	_	Extraction time, ultrasonic power, W/M ratio	0.26	Liu et al. (2019)
Lemon waste	OAHD^d^	_	W/M, extraction time, voltage gradient	2.26	Tunç and Odabaş (2021)
*Mentha spicata* L.	HD	Enzyme	Process time, pH, enzyme content, temperature Agitation	~ 0.1	Saldaña‐Mendoza et al. (2022)
*Coriandrum sativum* L.	HD	NaCl	Time of HD	Not affected	Huzar et al. (2018)

^a^ Hydro‐distillation.

^b^ Microwave‐assisted hydro‐distillation.

^c^ Ultrasound‐assisted hydro‐distillation.

^d^ Ohmic‐assisted hydro‐distillation.

Ultrasonic pretreatment of EOs has recently attracted the attention of researchers. This technique can overcome some of the disadvantages of conventional HD. For example, the treatment can overcome the challenges of long extraction times and low‐efficiency problems (Chen et al., 2007). HD can increase EO yields, as demonstrated in the following research works: Liu et al., 2019 (Iberis Amara seeds); Chen, Sun, et al., 2020 (*Cinnamomum cassia* bark); Boubechiche et al., 2017 (Allium sativum); Zorga et al., 2020 (Celery Seeds); and Chen, Liu, et al., 2020 (leaves of *Perilla frutescens)*. In addition, the concentration of oxygenated compounds increase when this technique is used (Da Porto & Decorti, 2009). Carvone is one of the main components of SEO. The content of carvone (an oxygenated monoterpene) is increased under this treatment (Chen, Liu, et al., 2020; Damyeh et al., 2016). This component has a significant relation with the antioxidant activity (Younis & Beshir, 2004). The carvone to limonene ratio is one of the characteristics of SEO quality. Studies have shown that the ratio of caraway seeds in cases of ultrasonic treatments was shorter than the control. (Assami et al., 2012).

Cold or non‐thermal plasma has been used for its beneficial properties in agriculture. Enhancement of seed germination, decontamination, and soil remediation are some of these properties. Its efficiency for microbiological safety of plant and animal origin foods is also undeniable (Misra et al., 2016; Ziuzina & Misra, 2016). The effect of dielectric barrier discharge cold plasma (DBD‐CP) has been extensively studied for several types of EOs: In fennel seeds and spearmint leaves, the EO yield improved under the optimized DBD‐CP treatment (Rezaei et al., 2021); in lemon peel oil extraction aided by DBD plasma treatment, the yield increased by 49.34%, although the EO constituents remained constant (Pragna et al., 2019); also, camelina seed oil extraction by DBD‐CP treatment showed a similar result. In general, studies have shown that the yield of oil extracted is positively affected by DBD‐CP. The protein content obtained by this treatment was also the largest (Rezaei et al., 2020). These researches show the positive aspects of CP or DBD‐CP application.

The above articles and research studies reveal the important effects of ultrasonic treatment and CP in addition to the varied performance of the extraction process in different water to plant ratios. To develop knowledge in this field, in this study, these treatments were tested together by RSM‐designed experiments to determine their effects on the amount of extracted EOs. The optimal extraction conditions of EO as a result of the interaction of these factors are determined and the effect of these interactions on the composition of essential oils is also evaluated. To design these experiments, a CCD design of RSM was used.

## 2 MATERIAL AND METHODS

### 2.1 Plant material preparation

Spearmint plants were cultivated at the research farm of the Faculty of Agriculture, Tarbiat Modares University. The samples were harvested in the vegetative stage. Finally, the plants were shade dried in the drying room at 25°C for 1 week.

### 2.2 Experimental design

For the design of the experiment, the design expert software version 13 was used. The CCD method of RSM is commonly used in optimization experiments, including a full or fractional factorial design. A group of star points improved the center points of the design (Danh et al., 2009). In this study, the randomized CCD was utilized. The number of numeric factors in UB was two (UB temperature and processing time). In W/M‐UB, three numeric factors were the W/M ratio, UB temperature, and UB processing time. In the third design, CP power, UB temperature, and UB processing time were the numeric factors.

The independent variables had five levels (Table 2a‐c) for each of the individual coded values. The runs contained no blocks for all three experiment designs. Six center points were applied for W/M‐UB and CP‐UB. The third case, that is, UB design, had 5 center points (Tables 3, 4, and 5).

**TABLE 2:** CCD five levels of each independent variable of (a) UB, (b) W/M‐UB, (c) CP‐UB

Factors	Minimum (−a)	Maximum (+a)	Coded low (−1)	Coded high (+1)	Mean
(a)					
UB temperature	30	80	37.32	72.68	54.23
UB time	1	30	5.25	25.75	15.45
(b)					
W/M	10	40	16.08	33.92	25
UB temperature	30	80	40.13	69.87	55
UB time	1	30	6.88	24.12	15.5
(c)					
CP power	15	24	16.82	22.18	19.25
UB temperature	30	80	40.13	69.87	55
UB time	1	30	6.88	24.12	15.5

**TABLE 3:** UB pre‐treatment independent variables and experimental yield of CCD

Run	UB temperature (°C)	UB time (min)	EO yield (%)
1	55	15.5	0.55
2	55	1	0.52
3	55	30	0.61
4	55	15.5	0.57
5	80	15.5	0.44
6	70	5	0.43
7	70	25.7	0.55
8	35	25.7	0.61
9	55	15.5	0.62
10	35	5	0.68
11	55	15.5	0.57
12	30	15.5	0.73
13	55	15.5	0.59

**TABLE 4:** W/M‐UB pretreatment independent variables and experimental yield of CCD

Run	W/M (ml/g)	UB temperature (°C)	UB time (min)	EO yield (%)
1	25	55	15.5	0.84
2	25	80	15.5	1.04
3	16	70	7	0.9
4	34	70	7	1.26
5	16	70	24	0.66
6	25	55	15.5	0.91
7	25	55	30	0.82
8	25	55	15.5	0.51
9	34	70	24	0.85
10	25	55	1	0.81
11	16	40	7	0.68
12	25	55	15.5	0.83
13	16	40	24	0.01
14	34	40	24	0.38
15	10	55	15.5	0.81
16	25	30	15.5	0.29
17	34	40	7	0.55
18	40	55	15.5	0.55
19	25	55	15.5	0.86
20	25	55	15.5	0.84

**TABLE 5:** CP‐UB pretreatment independent variables and experimental yield of CCD

Run	CP power (kV)	UB temperature (°C)	UB time (min)	EO yield (%)
1	22	70	24	0.65
2	24	55	15.5	0.78
3	19	80	15.5	0.53
4	19	55	15.5	0.62
5	19	55	15.5	0.63
6	17	70	24	0.51
7	19	55	30	0.77
8	17	70	7	0.44
9	22	40	24	0.95
10	22	40	7	0.67
11	19	30	15.5	0.65
12	19	55	15.5	0.63
13	19	55	1	0.49
14	19	55	15.5	0.57
15	17	40	24	0.69
16	22	70	7	0.6
17	19	55	15.5	0.59
18	17	40	7	0.46
19	19	55	15.5	0.63
20	15	55	15.5	0.59

### 2.3 UB pretreatment

The UB pretreatment was applied using an Elmasonic S 60 H UB processor (operating at 220–240 V). The samples were prepared under similar conditions to those used for UB pretreatment. Different W/M ratios were considered for W/M‐UB. For CP‐UB pretreatment, a variety of CP power values were applied as shown in Tables 3, 4, and 5. For change of the W/M ratio, 500 cc of water was added to the dry samples of weight 50, 31.25, 20, 14.7, and 12.5 g for ratios of 10, 16, 25, 34, and 40. The mixtures were treated at different UB temperatures and operating times (as shown in Tables 3, 4, and 5). Afterward, the treated samples were extracted by HD.

### 2.4 Cold plasma procedure

The CP pretreatment was enforced using a dual power supply CP reactor (Marzdashty et al., 2017). Dimensions of the device were 70 (w) × 45 (d) × 60 (h) cm and reactor characteristics were as follows: power 1: 0–25 kV, 50 Hz; and power 2: 0–10 kV, 6 kHz. The device contained two parallel electrodes. One of these (the lower) was stainless steel and was connected to a glass bowl (quartz) to produce homogenous CP at the various discharge gaps. This electrode was linked to a 50 Hz and variable voltage up to approximately 25 kV, while the second was linked to 6 kHz and 10 kV. The dried spearmint leaf samples (30 g each sample) were placed in the chamber and treated at different applied voltage values, while time durations for the treatments remained constant (5 min).

### 2.5 Essential oil isolation

The EO of treated samples was immediately extracted by the HD method. For the extraction, 30 g of dried spearmint were hydro‐distillated by a Clevenger type apparatus. The time duration of each sample for HD was 3 h. The EOs were collected in vials, dehydrated with anhydrous sodium sulfate and preserved in a refrigerator (4°C) until GC analysis.

### 2.6 Gas chromatography (GC) and gas chromatography–mass spectrometry (GC–MS)

Essential oil constituents were analyzed by gas chromatography (Agilent 7890B). The Agilent 7890B set‐up was coupled to a flame ionization detector and an HP‐5 capillary column (length 30 m, internal diameter 0.25 mm, and 0.25 μm film thickness). The temperature program was covered for 2 min at 60°C and then raised to 280°C at the rate of 5°C/min. GC–MS analysis was performed by a Thermoquest–Finnigan gas chromatograph equipped with a fused silica capillary HP‐5 column (60 m × 0.25 mm; i.e. film thickness 0.25 μm) coupled with a trace mass spectrometer. The flow rate of helium gas as the carrier was 1.1 ml/min in a split ratio of 1:100. The ionization voltage was 70 eV. Ion source and interface temperatures were 200 and 280°C. Mass range was adjusted from 45 to 456 amu. The same GC oven temperature program was used.

The EO constituents were identified by matching each component's mass spectra with those of the internal mass spectra library of the main library, Wiley 7.0 and Adams. Further identification was based on a comparison of peak retention indexes by using a homologous series of normal alkanes (C8 to C24) verified under the same operating situations and data published in the literature (Adams, 2007).

## 3 RESULTS AND DISCUSSION

### 3.1 Effect of process factors on EO extraction efficiency

In this study, for UB design, the effects and interaction of UB temperature and time were studied. The interaction of the treatments with W/M ratio factors were subjected to W/M‐UB design. In the third experiment design, the CP power interaction with UB factors was the numeric factor. The CCD fixed each independent variable at five levels. The levels were as follows: UB temperature: 30, 40, 55, 70, and 80°C, UB time duration: 1, 7, 15.5, 24, and 30, W/M ratio: 10, 16, 25, 34, and 40 and CP power: 15, 17, 19, 22, and 24 kV. The goal response was yield optimization with a maximizing EO target (Tables 3, 4, and 5). As shown, the SEO yields were found to be 0.43% w/w (UB temperature: 70°C and UB time: 5 min) to 0.73 (UB temperature: 30°C and UB time: 15.5 min) in UB pre‐treatment (Table 3). The yield was 0.01 (W/M ratio: 16, UB temperature: 40°C and UB time: 24 min) to 1.26 (W/M ratio: 34, UB temperature: 70°C and UB time: 7 min) in W/M‐UB results (Table 4). In CP‐UB 0.44% w/w (CP power: 17 kV, UB temperature: 70°C and UB time: 7 min) to 0.95% w/w (CP power: 22 kV, UB temperature: 40°C and UB time: 24 min) was the obtained yield (Table 5).

For the experimental data of UB pretreatment, a 2FI model was fitted, and the following equation was obtained for the treated EOs yield, predicted *R* ^2^ = 0.7773 and adjusted *R* ^2^ = 0.9002:

(1) Y=+0.5708–0.0891A+0.0256B+0.0483AB,

where *Y* is the EO percentage, *A* is the UB temperature, and *B* is the UB time.

For the experimental data of W/M‐UB pretreatment, a linear model was fitted, and the following equation was obtained for the treated EOs yield, predicted *R* ^2^ = 0.8263 and adjusted *R* ^2^ = 0.8754:

(2) Y=+0.6980+0.0619A+0.2603B−0.1663C,

where *Y* is the EO percentage, *A* is the water to material ratio, *B* is the UB temperature, and *C* is the UB time.

**FIGURE 1:**
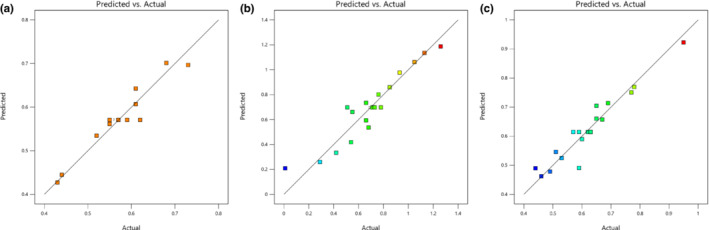
(a) Comparison actual values with predicted values of Ultrasonic Bath model. (b) Comparison actual values with predicted values of the water/material‐Ultrasonic Bath model. (c) Comparison actual values with predicted values of Cold Plasma‐Ultrasonic Bath model.

Also for the experimental data of CP‐UB pretreatment, a 2FI model was fitted. The resulting equation for the treated EOs yield is as follows, predicted *R* ^2^ = 0.7584 and adjusted *R* ^2^ = 0.8780:

(3) $$\begin{array}{c} Y=+0.6302+0.0828A-0.0582B+0.0816C-0.0252\mathrm{AB}\\ \;+0.0037\;\mathrm{AC}-0.0490\;\mathrm{BC}, \end{array}$$

where *Y* is the EO percentage, *A* is the CP power, *B* is the UB temperature, and *C* is the UB time. Tables 6a‐c displayed the results of ANOVA for the models, and *F*‐values of 37.09, 45.48, and 23.8 indicated that the models are significant. In the model of UB pretreatment, A, B, and AB, in W/M‐UB pretreatment, A, B, and C, and in the third model, A, B, C, and BC were the significant terms. The lack of fit *F*‐values of 0.9816, 1.19, and 3.6 show that the lack of fit is not significant relative to the pure error. The model's *p*‐values were less than .05 indicating that the models are significant. Figure 1a–c showed that data points corresponding to predicted and actual values are scattered across the 45‐degree line with proximity, suggesting that the actual findings are precisely predicted from the factor values.

**TABLE 6:** ANOVA for 2FI model of (a) UB pretreatment, (b) W/M‐UB pretreatment, (c) CP‐UB pretreatment

Source	Sum of squares	df	Mean square	*f*‐Value	*p*‐value	Remarks
(a)						
Model	0.0771	3	0.0257	37.09	<.0001	Significant
A^a^	0.0633	1	0.0633	91.30	<.0001	Significant
B^b^	0.0052	1	0.0052	7.54	.00226	Significant
AB	0.0094	1	0.0094	13.56	.0051	significant
Residual	0.0062	9	0.0007	‐	‐	‐
Lack of fit	0.0034	5	0.0007	0.9816	.5217	Non‐significant
Pure error	0.0028	4	0.0007	‐	‐	‐
Core total	0.0833	12	‐	‐	‐	‐
(b)						
Model	1.36	3	0.4530	45.48	<.0001	Significant
A^c^	0.0529	1	0.0529	5.31	.0350	Significant
B^a^	0.9349	1	0.9349	93.85	<.0001	Significant
C^b^	0.3713	1	0.3713	37.27	<.0001	Significant
Residual	0.1594	16	0.0100	‐	‐	‐
Lack of fit	0.1152	11	0.0105	1.19	.4532	Non‐significant
Pure error	0.0441	5	0.0088		‐	‐
Core total	1.52	19	‐	‐	‐	‐
(c)						
Model	0.2440	6	0.0407	23.80	<.0001	Significant
A^d^	0.0878	1	0.0878	51.36	<.0001	Significant
B^a^	0.0463	1	0.0463	27.08	.0002	Significant
C^b^	0.0883	1	0.0883	51.70	<.0001	Significant
AB	0.0046	1	0.0046	2.68	.1253	Non‐significant
AC	0.0001	1	0.0001	0.0555	.8175	Non‐significant
BC	0.0190	1	0.0190	11.13	.0054	Significant
Residual	0.0222	13	0.0017	‐	‐	‐
Lack of fit	0.0189	8	0.0024	3.60	.0871	Non‐significant
Pure error	0.0033	5	0.0007	‐	‐	‐
Core total	0.2662	19	‐	‐	‐	‐

^a^ UB temperature.

^b^ UB time.

^c^ W/M.

^d^ CP power.

### 3.2 Essential oil yield optimization

#### 3.2.1 Essential oil yield optimization as a function of UB factors interaction

**FIGURE 2:**
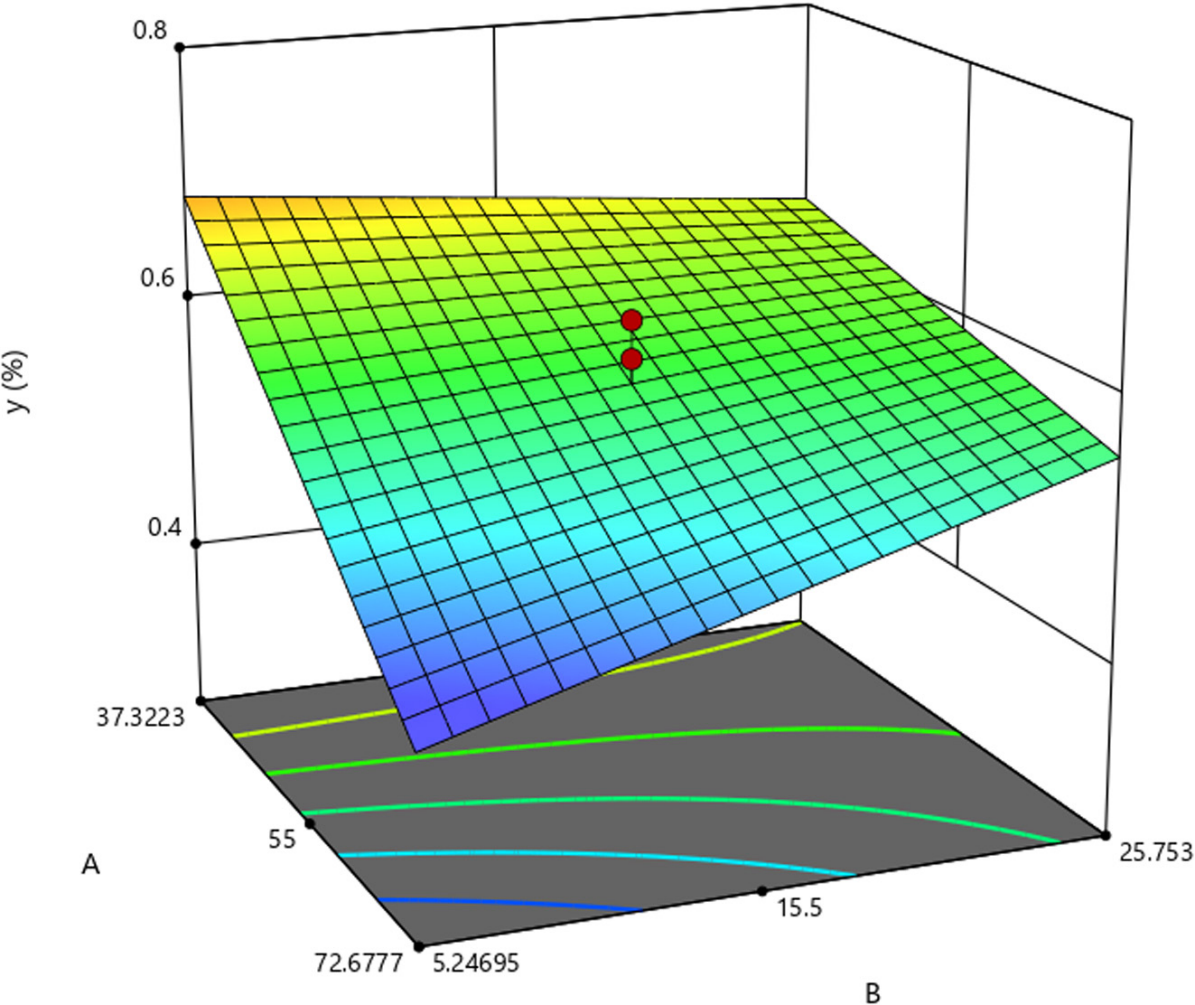
Response surface plot showing the effect of UB temperature (A) and UB time (B) on EO percentage (Y).

Figure 2 shows the EO yield as a function of UB time and UB temperature and reveals that, as a general rule, UB had a positive effect on the EO extraction efficiency. However, the UB temperature had an inverse relationship with EO yield.

The optimization of EO extraction as a function of UB temperature and time duration, to maximize the EO extraction efficiency, was done. The consequent optimized conditions were as follows: UB temperature: 37.3°C and UB time: 5.2 min.

Ultrasonic treatment has been shown to result in higher levels of EO extraction in cinnamon bark (Chen, Sun, et al., 2020), *Iberis Amara* seeds (Liu et al., 2019), citronella grass (Kumoro et al., 2021), and kumquat peel (Yu et al., 2021) in other studies.

#### 3.2.2 Essential oil yield optimization as a function of W/M‐UB factors interaction

**FIGURE 3:**
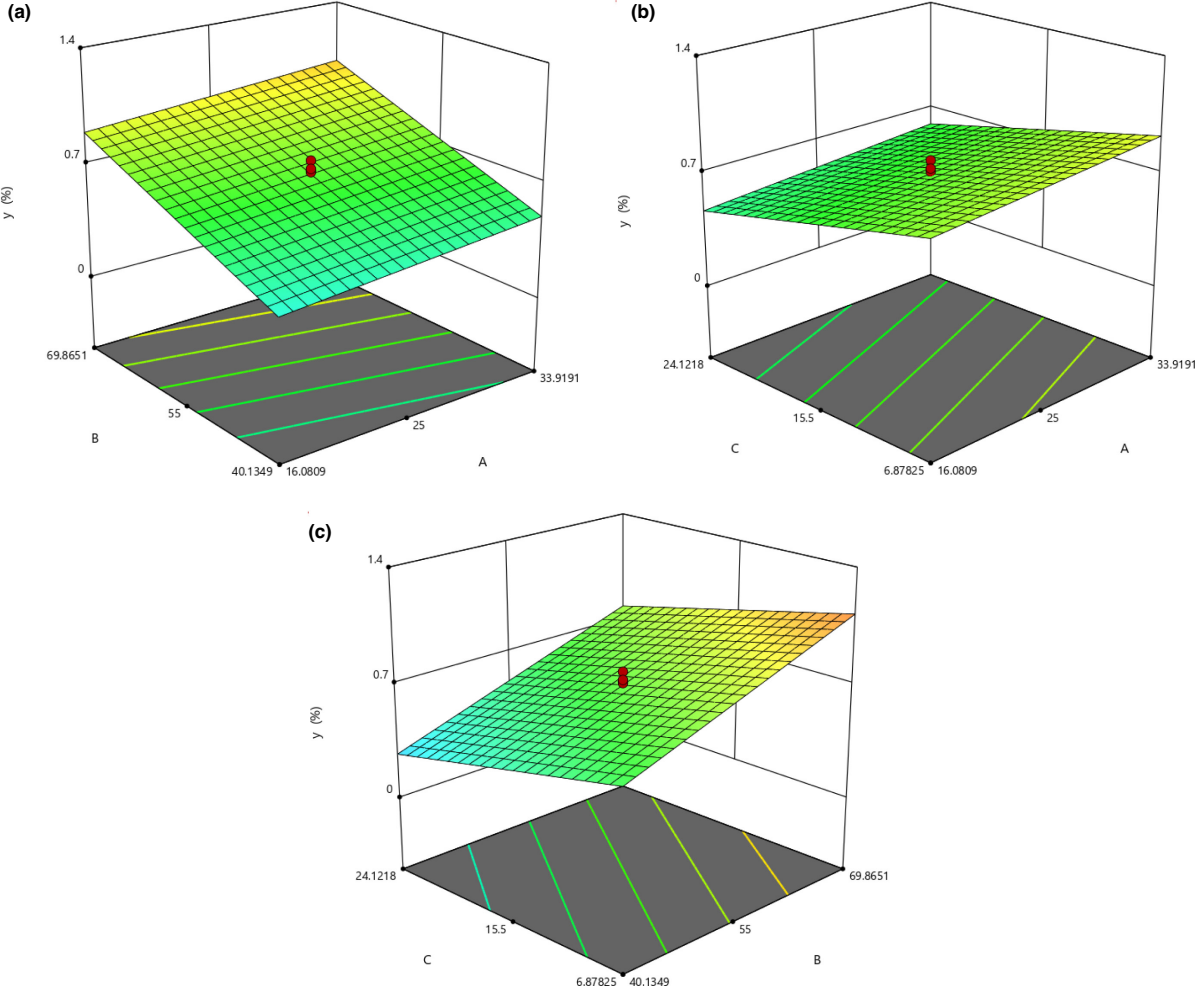
Response surface plot showing (a) the effect of W/M ratio (A) and UB temperature (B) on EO percentage (Y) at fixed UB time of 15.5 min. (b) the effect of W/M ratio (A) and UB time (C) on EO percentage (Y) at a fixed UB temperature of 55°C and (c) the effect of UB temperature (B) and UB time (C) on EO percentage (Y) at fixed W/M ratio of 25.

Figure 3 illustrates the EO percentage as a function of W/M‐UB factors. From Figure 3, it can be understood that generally, the increase in W/M ratio leads to more extraction of EO after HD (Figure 3a,b). Figure 3c shows the effect of UB factors on EO percentage. From Figure 3c, it can be inferred that increasing UB temperature and decreasing UB time treatment improves SEO yield.

The optimized conditions as a result of the interaction of the factors are 33.9 of W/M ratio, 69.9°C of UB temperature, and 6.9 min of UB time.

#### 3.2.3 Essential oil yield optimization as a function of CP‐UB factors interaction

The optimization of EO extraction as a function of CP power, UB temperature, and UB time duration, to maximize the EO extraction efficiency was proven and was found to be as follows: CP power: 22.176, UB temperature: 40.135, and UB time: 24.122.

**FIGURE 4:**
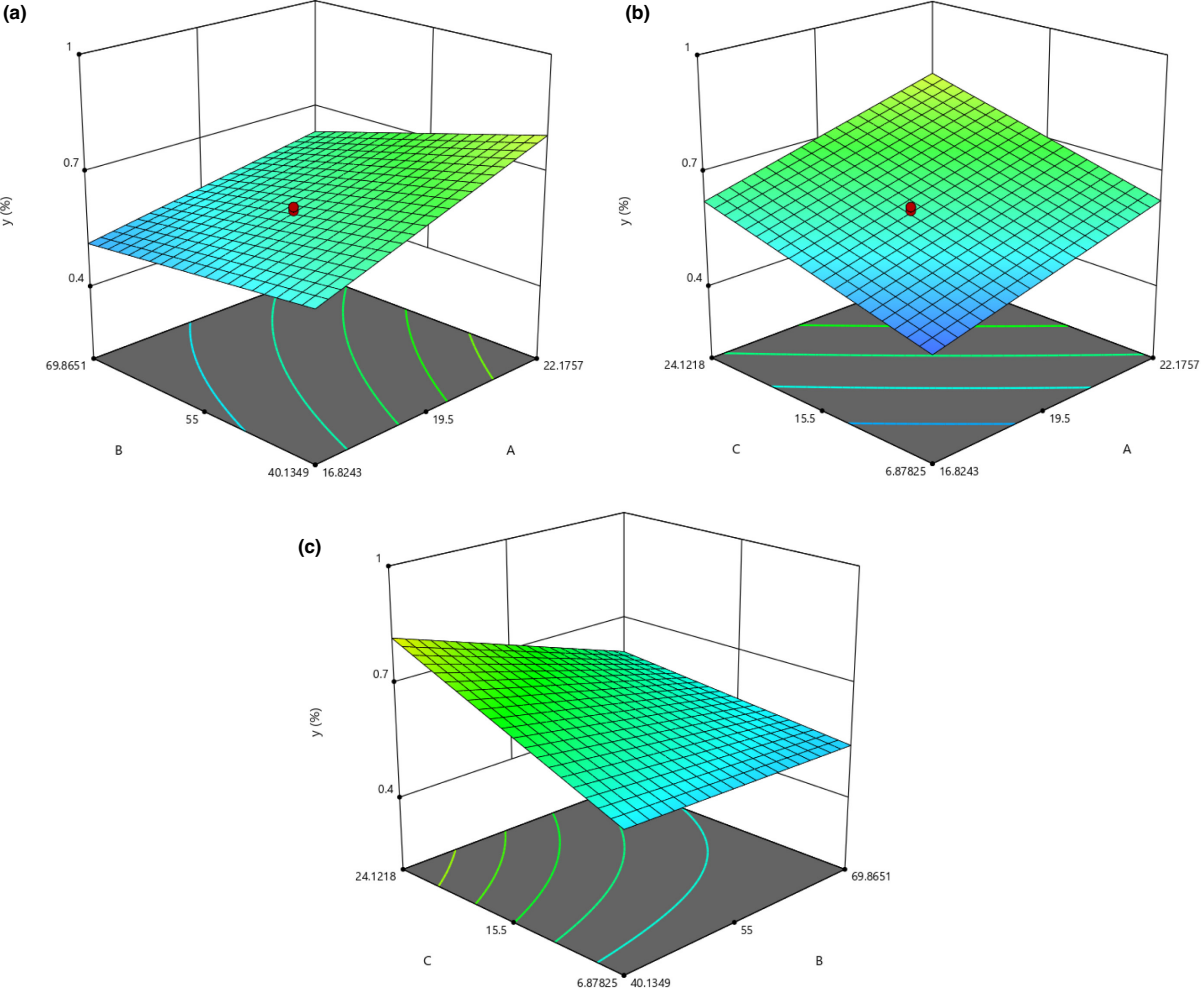
Response surface plot showing (a) the effect of CP power (A) and UB temperature (B) on EO percentage (Y) at fixed UB time of 15.5 min. (b) the effect of CP power (A) and UB time (C) on EO percentage (Y) at a fixed UB temperature of 55°C and (c) the effect of UB temperature (B) and UB time (C) on EO percentage (Y) at fixed CP power of 25 kV.

As shown in Figure 4a,b, a raise in CP power can improve the SEO yield after HD. As a result similar to other figures, UB temperature has an inverse effect on EO yield; however, UB time has a direct relationship with the SEO yield (Figures 3a–c and 4a–c).

For example, in other studies, in lemon peel (Pragna et al., 2019) and cumin seeds (Sharanyakanth et al., 2021), CP treatment followed by HD has been shown to improve EO extraction.

### 3.3 Effect of pretreatments on spearmint essential oil yield

Spearmint essential oil yield in the control sample (conventional HD) was 0.61%, whereas SEO percentages of 0.73%, 1.26%, and 0.95% were obtained from UB, W/M‐UB, and CP‐UB treatments. In other words, UB, W/M‐UB, and CP‐UB pretreatments in optimized conditions raised SEO yield to 119.7%, 206.6%, and 155.7% in comparison to the control sample (Figure 6).

### 3.4 Effect of pretreatments on EO composition

**FIGURE 5:**
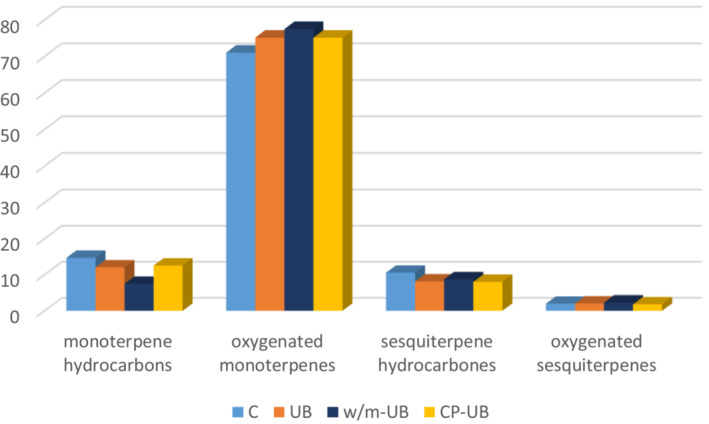
Comparison of EO constituent’s classes in control and treated samples (C: control).

As shown in Table 7 and Figure 5, analysis of the EO resulted in the identification of 45 compounds. Oxygenated monoterpenes (from 71.07% in control to 77.52% in W/M‐UB treated sample) represented 96%–98.32% of the total EO, followed by monoterpene hydrocarbons (from 7.46% in W/M‐UB treated to 14.71% in control sample). This balance of the major components is in agreement with previous research (Rezaei et al., 2021). Carvone, limonene, dihydrocarveol <fneo‐>, cineole <1,8‐>, linalool, piperitenone, and menthone <iso‐> were the main components in all samples.

**TABLE 7:** Constituents of EOs in control and treated samples

No.	Compounds	RI	Yield (%)			
			C	UB	W/M‐UB	CP‐UB
1	Pinene <α‐>	932	0.97	0.69	0.47	0.7
2	Camphene	946	0.18	0.15	0.24	0.15
3	Sabinene	969	0.57	0.51	0.34	0.51
4	Pinene <β‐>	974	1.77	1.4	0.81	1.43
5	Myrcene	988	0.59	0.47	0.34	0.48
6	Limonene	1024	10.28	8.64	5.26	8.96
7	Cineole <1,8‐>	1026	5.14	5.06	4.14	5.6
8	Terpinene <γ‐>	1054	0.14	‐	‐	0.13
9	Terpinolene	1086	0.21	0.2	‐	0.19
10	Linalool	1095	4.2	5.24	2.4	3.72
11	Octanol acetate <3‐>	1120	0.22	0.22	‐	0.22
12	Menthone	1148	0.82	0.78	1.59	0.92
13	Menthone <iso‐>	1158	3.57	4.02	22.05	3.45
14	Menthofuran	1159	1.7	1.79	2.89	1.93
15	Borneol	1165	0.53	0.67	0.76	0.61
16	terpinene‐4‐ol	1174	‐	‐	0.44	‐
17	Terpineol <α‐>	1186	‐	‐	0.19	‐
18	Dihydro carveol <neo‐>	1193	7.65	7.73	6.33	8.51
19	Caranone <cis‐4‐>	1200	0.23	0.51	0.16	0.21
20	Carveol <cis‐>	1226	0.6	0.53	0.33	0.58
21	Pulegone	1233	2.37	2.73	3.13	2.41
22	Carvone	1239	36.95	38.93	27.77	40.05
23	Piperitone	1249	0.42	0.47	0.34	0.42
24	Isopulegyl acetate <neo‐>	1274	0.13	0.15	‐	0.12
25	Isobornyl acetate	1283	0.16	0.17	0.13	0.17
26	Dihydro carveol acetate <neo‐>	1306	0.18	0.19	0.17	0.19
27	Dihydro carveol acetate <iso‐>	1326	1.55	1.46	1.35	1.59
28	Piperitenone	1340	3.96	3.9	2.84	3.78
29	Carvyl acetate <cis‐>	1365	0.48	0.44	0.34	0.5
30	Isoledene	1374	1.03	0.81	1	0.83
31	Bourbonene <β‐>	1387	3.37	3.57	2.14	3
32	Thujaplicin <α‐>	1410	0.21	0.2	0.17	0.2
33	Caryophyllene <(E)‐>	1417	2.98	2.42	3.07	2.52
34	Gurjunene <β‐>	1431	0.2	0.16	0.18	‐
35	Humulene <α‐>	1452	0.21	0.25	0.3	0.16
36	Cadina‐1(6), 4‐diene <trans‐>	1475	0.92	0.57	0.78	0.74
37	Muurolene <γ‐>	1478	0.2	‐	0.12	0.15
38	Himachalene <γ‐>	1481	0.3	0.16	0.51	0.26
39	Muurola‐4(14), 5‐diene <trans‐>	1493	‐	‐	0.2	‐
40	Bicyclogermacrene	1500	0.2	‐	‐	0.16
41	Cadinene <γ‐>	1513	‐	‐	0.24	‐
42	Calamenene <cis‐>	1528	1.16	0.19	0.2	0.2
43	Spathulenol	1577	1.42	1.48	1.71	1.46
44	Atlantol <β‐>	1608	0.34	0.34	0.34	0.35
45	Torreyol	1644	0.21	0.21	0.23	0.22
	Total		98.32	97.41	96	97.78

In the optimized W/M‐UB treatment, Menthone <iso‐> content is significantly higher than control and other treated samples; in contrast, carvone percent in W/M‐UB treatment is considerably lower in comparison with other EOs (Table 7).

Spearmint essential oil is characterized by the dominant presence of carvone. The carvone content found here was from 27.77 in W/M‐UB to 40.05 in CP‐UB. The dominance of carvone has also been shown by other researchers such as Yang et al., 2021, who found it at 52.25%; Chrysargyris et al., 2019, who found it varied from 61.31% in 100 Mm salinity NaCl to 72.87% in control sample EO; and Farahbakhsh et al., 2021, who identified carvone occurring at 49.91%. The carvone content in UB and CP‐UB treatments was higher than control but the W/M‐UB treatment diminished the carvone percentage.

**FIGURE 6:**
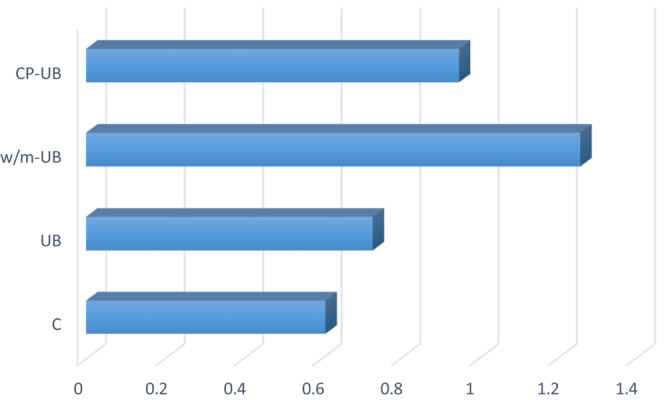
SEO yield (%) in control and treated samples (C: control).

CP‐UB pretreatment increased the yield of carvone, dihydrocarveol <neo‐>, and cineole <1,8‐>. In addition, CP‐UB resulted in the reduction of limonene, linalool, piperitenone, and menthone <iso‐>. Thr carvone percentage in the treatment was higher than other EO samples. There is a direct correlation between the EO yield and the percentage yield of oxygenated monoterpenes. Additionally, SEO's high level of monoterpenes shows high antifungal and enzyme inhibitory activities (Ali‐Shtayeh et al., 2019). As shown in Figure 5, oxygenated monoterpenes yield in all treated samples was higher than control. On the other hand, the EO percentage in treated samples was higher than in the control (Figure 6).

## 4 CONCLUSION

Spearmint essential oil is one of the most widely used and valuable EOs on the world market. Many studies have been performed on different methods of EO extraction and its compounds. The results of this study revealed that SEO extraction can be raised by applying UB, W/M‐UB, and CP‐UB pretreatments. Although the interaction of the applied treatments led to the optimal conditions of UB temperature 37.3°C and UB time 5.2 min for UB design. The optimal conditions were the W/M‐UB 33.9 of W/M ratio, 69.9°C of UB temperature, and 6.9 min of UB time. The following conditions were also obtained as optimal conditions for CP‐UB: CP power: 22.176, UB temperature: 40.135, and UB time: 24.122. Three‐dimensional graphs of UB time‐UB temperature‐EO yield and CP power were also evaluated. The graphs showed that increasing the time duration of UB and the power of CP led to an increase in the amount of extracted EO, although increasing the UB temperature led to a decrease in the amount of extracted EO. Additionally, the W/M ratio had an inverse correlation with the SEO yield. The content of oxygenated monoterpenes in all three optimized conditions was slightly higher than in the control, although monoterpene hydrocarbon percentages were lower than the control in all optimized conditions. Menthone <iso‐> at W/M‐UB optimized conditions was higher than control. The carvone percentage in the condition was lower in comparison to control and other optimized models. But the carvone to limonene ratio as a qualitative indicator in the treated samples (4.51 and 4.47 in UB and CP‐UB, respectively) was higher than the control (3.59). The carvone to limonene ratio indicated that the applied treatments improved the SEO quality. The amount of extracted EO in the optimized samples was 119.7%, 206.6%, and 155.7% in optimized conditions of UB, W/M‐UB, and CP‐UB, in comparison to the control sample. In addition, these treatments may improve antifungal and enzyme inhibitory properties. Further research and study of the economic aspects of these treatments on an industrial scale may help the EO and herbal medicine industries.

## FUNDING INFORMATION

We are thankful to the Tarbiat Modares University (TMU) for funding this research.

## CONFLICT OF INTEREST

The authors declare that they have no conflict of interest.

## ETHICAL STATEMENT

“This study does not involve any human or animal testing”.

## INFORMED CONSENT

Written informed consent was obtained from all study participants.

## Data Availability

All data generated or analyzed during this study are included in this published article.
